# Pre-clinical efficacy and synergistic potential of the MDM2-p53 antagonists, Nutlin-3 and RG7388, as single agents and in combined treatment with cisplatin in ovarian cancer

**DOI:** 10.18632/oncotarget.9499

**Published:** 2016-05-20

**Authors:** Maryam Zanjirband, Richard J. Edmondson, John Lunec

**Affiliations:** ^1^ Northern Institute for Cancer Research, Newcastle University, Newcastle upon Tyne NE2 4HH, United Kingdom; ^2^ Faculty Institute for Cancer Sciences, University of Manchester, Manchester M13 9WL, United Kingdom

**Keywords:** ovarian cancer, MDM2-p53 antagonists, Nutlin-3/RG7388, p53, combined treatment

## Abstract

Ovarian cancer is the fifth leading cause of cancer-related female deaths. Due to serious side effects, relapse and resistance to standard chemotherapy, better and more targeted approaches are required. Mutation of the *TP53* gene accounts for 50% of all human cancers. In the remaining malignancies, non-genotoxic activation of wild-type p53 by small molecule inhibition of the MDM2-p53 binding interaction is a promising therapeutic strategy. Proof of concept was established with the cis-imidazoline Nutlin-3, leading to the development of RG7388 and other compounds currently in early phase clinical trials. This preclinical study evaluated the effect of Nutlin-3 and RG7388 as single agents and in combination with cisplatin in a panel of ovarian cancer cell lines. Median-drug-effect analysis showed Nutlin-3 or RG7388 combination with cisplatin was additive to, or synergistic in a p53-dependent manner, resulting in increased p53 activation, cell cycle arrest and apoptosis, associated with increased p21^WAF1^ protein and/or caspase-3/7 activity compared to cisplatin alone. Although MDM2 inhibition activated the expression of p53-dependent DNA repair genes, the growth inhibitory and pro-apoptotic effects of p53 dominated the response. These data indicate that combination treatment with MDM2 inhibitors and cisplatin has synergistic potential for the treatment of ovarian cancer, dependent on cell genotype.

## INTRODUCTION

Ovarian cancer is the most lethal of all gynecological malignancies and was reported to be responsible for approximately 152,000 deaths worldwide in 2012 [1]. Although patients with primary disease respond to platinum and taxane chemotherapy, relapse and resistance to treatment is prevalent, leading to lack of long-term benefit from treatment. For this reason, molecular alterations in tumors, particularly those involved in growth signaling pathways, cell cycle progression and apoptosis are being investigated to potentially exploit for targeted therapy (reviewed by [2, 3]).

The *TP53* tumor suppressor gene is referred to as the most frequently altered gene in human cancers. It has been substantially established that p53 protects cells against environmental and intra-cellular stress stimuli by playing a central role in regulating cell cycle control, differentiation, proliferation, DNA repair and apoptosis (reviewed by [4]). *TP53* mutation is the most frequent genetic abnormality in ovarian cancer, which accounts for 60% of ovarian cancers, with a particularly high prevalence in high grade serous tumors. In the remaining malignancies, p53 function is held in check through other mechanisms and reactivation of p53 is a potential therapeutic strategy (reviewed by [5]).

MDM2 is the main negative regulator of p53, regulating p53 through ubiquitin dependent degradation. The imidazoline Nutlin-3, was the first non-genotoxic specific small-molecule antagonist of the MDM2-p53 binding interaction to be developed [6] and has been used extensively as a probe compound in preclinical and mechanistic studies. RG7388 was subsequently developed as a second generation MDM2 inhibitor with superior potency, selectivity and oral bioavailability suitable for clinical development to inhibit the MDM2-p53 interaction and activate the p53 pathway [7, 8]. These compounds target a small hydrophobic pocket on MDM2, to which p53 normally binds, leading to p53 stabilization and upregulation of p53 downstream transcriptional targets involved in cell cycle arrest and/or apoptosis, including genes encoding p21^WAF1^, BAX and BBC3 (PUMA) [9, 10]. Using MDM2-p53 antagonists as single-agent therapy has been suggested to be potentially limited due to acquisition of resistance through continuous exposure to MDM2 inhibitors followed by *de novo* mutations [11] and (reviewed by [12]). It is therefore logical to consider using MDM2 antagonists in combination with established therapeutic agents to improve treatment with the possibility of dose reduction and less normal tissue cytotoxicity and genotoxicity. In the context of ovarian cancer it is of interest to investigate the combination of cisplatin and MDM2 inhibitors, particularly as individually these agents have different dose limiting toxicities.

The aim of the present study was to test a panel of established ovarian cancer cell lines for their response to MDM2-p53 antagonists, Nutlin-3 and RG7388, alone and in combination with cisplatin and examine the mechanistic basis of these responses in relation to the genotype and induced gene expression of the cells.

## RESULTS

### Wild-type *TP53* ovarian cancer cell lines are sensitive to Nutlin-3/RG7388

**Figure 1:**
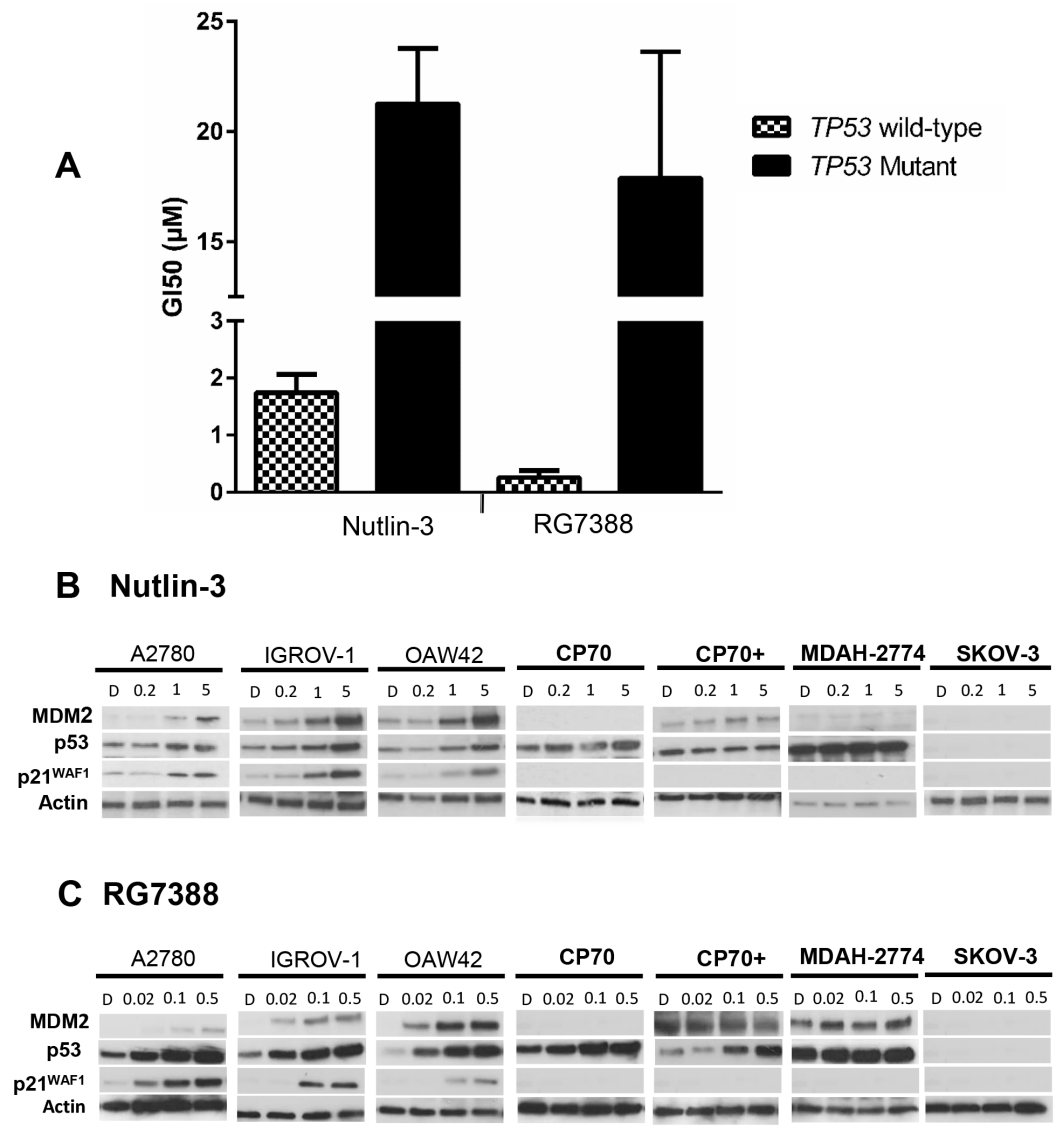
The sensitivity to MDM2 antagonists, Nutlin-3 and RG7388, in a panel of wild-type and mutant *TP53* ovarian cancer cell lines **A.** Wild-type *TP53* cell lines are significantly more sensitive to growth inhibition by Nutlin-3 (Mann Whitney test, p< 0.0001) and RG7388 (Mann Whitney test, p< 0.0001) treatment for 72 hours compared to mutant *TP53* cell lines. Data shown are the average of at least three independent experiments and error bars represent SEM. Western blot analysis for **B.** Nutlin-3 and **C.** RG7388 showed stabilization of p53 and upregulation of p53 transcriptional target gene protein levels, MDM2 and p21^WAF1^, four hours after the commencement of treatment in wild-type *TP53* cell lines with the indicated doses (μM); however, they had no effect on downstream transcriptional targets of p53 in mutant *TP53* ovarian cancer cell lines with the delivered dose range of MDM2 antagonists despite stabilization of the mutant p53 in the CP70 +and CP70+ cells. *TP53* mutant cell lines are highlighted in bold font. **D.** DMSO treated control cells; CP70+, *MLH1- Corrected CP70+*.

Growth inhibition by Nutlin-3/RG7388 was investigated using the sulforhodamine-B (SRB) assay for a panel of wild-type and mutant *TP53* ovarian cancer cell lines derived from tumours of different histological subtypes [13–16] (Figure 1A and Table 1). The required concentration of each compound leading to 50% growth inhibition (GI_50_) showed that wild-type *TP53* ovarian cancer cell lines were significantly more sensitive to Nutlin-3/RG7388 compared to mutant, which is consistent with their mechanism of action (*p<0.0001* Mann-Whitney test). Also, RG7388 was more potent compared to Nutlin-3 (*p<0.0001* Mann-Whitney test). The GI_50_ values for wild-type *TP53* cell lines for RG7388 and Nutlin-3 were in the nanomolar range (253.3 ± 73.1 (SEM) nM) and micromolar range (1.76 ± 0.51 (SEM) μM) respectively. In contrast, *TP53* mutant cell lines had GI_50_ values greater than 10 μM (17.8 ± 2.9 (SEM) μM) for RG7388 and range 21.2->30 μM for Nutlin-3 (Table 1 and Figure 1A).

**Table 1:** GI_50_ concentrations of cisplatin, Nutlin-3 and RG7388 for the panel of ovarian cancer cell lines of varying *TP53* status

Cell line	*TP53* status	Histotype & Reference	Cisplatin (μM)	Nutlin-3 (μM)	RG7388 (μM)
**A2780**	Wild-type	Undifferentiated [12]	0.82 ± 0.17	1.23 ± 0.23	0.11 ± 0.01
**IGROV-1**	Wild-type	Mixed, EC with CCC/UD [14]	0.85 ± 0.04	2.8 ± 0.48	0.35 ± 0.04
**OAW42**	Wild-type	Serous Cystadenocarcinoma [13]	0.73 ± 0.02	1.3 ± 0.1	0.31 ± 0.04
**CP70**	Mutant (Heterozygous) c.514 G->T; p.Val172Phe	Undifferentiated [12]	5.8 ± 1.1	21.2 ± 2.5	11.7 ± 1.81
***MLH1*-Corrected CP70+**	Mutant (Heterozygous) c.514 G->T;. Val172Phe	Undifferentiated [12]	2.4 ± 0.25	21.2 ± 1.22	14.5 ± 1.09
**MDAH-2774**	Mutant (Homozygous) c.818G->A; p.Arg273His	Endometrioid carcinoma [15]	1.11± 0.14	21.4 ± 0.9	20.7 ± 1.43
**SKOV-3**	Mutant (Homozygous) 265delC; p.Pro89fsX33	Adenocarcinoma [13]	8.8 ± 0.49	> 30	24.6 ± 1.54

Data represent the mean of at least 3 independent experiments ± SEM. EC, endometrioid carcinoma; CCC, clear cell carcinoma; UD, undifferentiated.

### Functional activation of the p53 pathway in wild-type *TP53* ovarian cancer cell lines in response to Nutlin-3/RG7388

The p53-dependent response to Nutlin-3/RG7388 assessed by Western blotting (Figure 1B, 1C) showed that Nutlin-3/RG7388 induced stabilization of p53 and upregulation of p21^WAF1^ and MDM2 protein levels four hours after the commencement of treatment in a concentration-dependent manner and confirmed functional activation of wild-type p53 by release from MDM2. However, as anticipated, it had no effect on p53-dependent gene expression in the *TP53*-mutant cell lines with the delivered dose range of Nutlin-3/RG7388. Interestingly this was despite a small increase in stabilization of mutant p53 in response to RG7388 at the doses of 0.1 and 0.5 μM with the CP70 and *MLH1*-corrected CP70+ cell lines. This result indicates that some forms of mutant p53 are still targeted for degradation by MDM2 even though they have lost their transcriptional function. Also, there is a frame-shift deletion in the SKOV-3 cell line (Table 1) leading to an absence of detectable p53, p21^WAF1^ and MDM2 expression (Figure 1B, 1C).

### Nutlin-3/RG7388 synergizes with cisplatin for growth inhibition of wild-type *TP53* ovarian cancer cell lines

**Figure 2:**
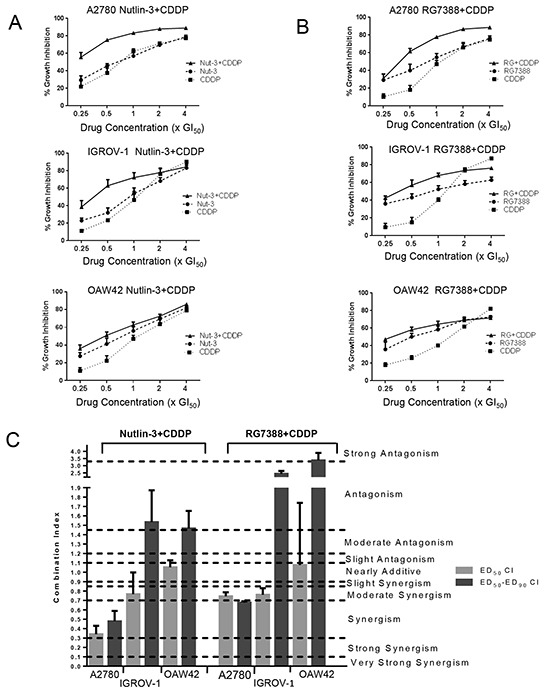
Nutlin-3/RG7388 synergizes with cisplatin in wild-type *TP53* ovarian cancer cells **A.** Growth inhibition curves of three wild-type *TP53* cell lines exposed to Nutlin-3 and CDDP alone, and in combination at constant 1:1 ratios of 0.25X, 0.5X, 1X, 2X and 4X their respective GI_50_ concentration for 72 hours. **B.** Growth inhibition curves of three wild-type *TP53* cell lines exposed to RG7388 and CDDP alone, and in combination at constant 1:1 ratios of 0.25X, 0.5X, 1X, 2X and 4X their respective GI_50_ concentrations for 72 hours. **C.** The CI values for Nutlin-3/RG7388 in combination with cisplatin at ED_50_ and the average of CI values at effect levels ED_50_, ED_75_ and ED_90_ in three wild-type *TP53* ovarian cancer cell lines. Data are shown as the average of at least 3 independent experiments and error bars represent SEM. Nut-3, Nutlin-3; RG, RG7388; CDDP, cisplatin; CI, Combination Index.

The effect of Nutlin-3/RG7388 in combination with cisplatin was investigated for 3 wild-type *TP53* ovarian cancer cell lines using Median-effect analysis. The sensitivity of these *TP53* wild-type cell lines to growth inhibition during 72 hours exposure to Nutlin-3, RG7388 and cisplatin was determined as single agents, and in combination at 5 equipotent concentrations between 0.25× and 4× their respective GI_50_ concentrations. The effect of combined treatment was cell type and compound dependent. Combination of Nutlin-3/RG7388 with cisplatin at all concentrations led to greater growth inhibition compared to either agent alone for the A2780 cell line. Combination treatment of OAW42 and IGROV-1 cell lines also produced more growth arrest at concentrations equal to or lower than the individual 1xGI_50_ dose (Figure 2A & 2B). To determine whether the observed differences in growth inhibition were additive or synergistic, the data were analyzed using median-effect analysis and Combination Index (CI) and Dose Reduction Index (DRI) values calculated.

CI values for each constant ratio combination and at effect levels of ED_50_, ED_75_ and ED_90_ were computed. Also, the average of CI values at ED_50_, ED_75_ and ED_90_ was determined (Table 2 and Figure 2C). Across all cell lines, the effect of combination treatment of Nutlin-3/RG7388 with cisplatin ranged from additive to synergistic based on the CI at ED_50_. Although the effect of combined treatment based on overall CI was synergism for A2780, it was antagonism for IGROV-1 and OAW42 (Table 2 and Figure 2C). The data analysis showed there was a favourable dose reduction index (DRI), which demonstrates how many-fold the dose of each drug in a combination treatment may be reduced to achieve a given effect level compared with the doses of each drug alone (Table 3). Both Nutlin-3, and RG7388 had favorable DRI values for combined treatment with cisplatin, with most experimental values ranging from 1.1-fold to 6.9-fold dose reduction. These DRI values have clinical implications, demonstrating a significant individual drug dose reduction may be achieved for a given combination therapeutic effect, compared with the dose of either drug alone as a single agent to obtain the same therapeutic effect.

**Table 2:** Growth inhibition CI values for RG7388/Nutlin-3 in combination with cisplatin for the wild-type *TP53* ovarian cancer cell lines

Cell Line	Combination	CI					CI ED_50_	CI ED_75_	CI ED_90_	CI Ave ED_50-90_
		XGI50								
		0.25	0.5	1	2	4				
**A2780**	Nut-3+CDDP	**0.5**	**0.4**	**0.4**	**0.6**	1.0	**0.3**	**0.5**	**0.8**	**0.5**
	RG+CDDP	1.2	**0.5**	**0.5**	**0.5**	**0.9**	**0.7**	**0.7**	**0.6**	**0.7**
**IGROV-1**	Nut-3+CDDP	**0.8**	**0.7**	1.0	1.6	2.2	**0.8**	1.4	2.5	1.5
	RG+CDDP	**0.8**	**0.8**	**0.7**	1.1	1.9	**0.8**	1.5	5.1	2.4
**OAW42**	Nut-3+CDDP	1.0	1.1	1.3	1.7	1.5	1.1	1.4	1.9	1.5
	RG+CDDP	1.3	1.2	1.5	2.1	3.1	1.1	3.5	5.6	3.4

The combined treatment was performed at the indicated fixed 1:1 ratios relative to their respective GI50 concentrations. CI values were calculated for each constant ratio combination and at effect levels ED50, ED75 and ED90 from the average of at least three independent experiments. CI Ave ED50-90 represents the average of CI values at effect levels of ED50, ED75 and ED90. CI range: < 0.1 very strong synergism; 0.1-0.3 strong synergism; 0.3-0.7 synergism; 0.7-0.85 moderate synergism; 0.85-0.9 slight synergism; 0.9-1.1 nearly additive; 1.1-1.2 slight antagonism; 1.2-1.45 moderate antagonism; 1.45-3.3 antagonism; 3.3-10 strong antagonism; > 10 very strong antagonism. Synergistic combinations are highlighted in bold font. Nut-3, Nutlin-3; RG, RG7388; CDDP, cisplatin; CI, Combination Index.

**Table 3:** DRI values for growth inhibition by RG7388/Nutlin-3 in combination with cisplatin for the wild-type ovarian cancer cell lines

Cell Line	Combination	Component	DRI				
			XGI50				
			0.25	0.5	1	2	4
**A2780**	Nut-3+CDDP	Nut-3	**2.6**	**5.7**	**5.5**	**4.3**	**2.6**
		CDDP	**4.3**	**6.9**	**6.4**	**5.2**	**3.2**
	RG+CDDP	RG7388	**1.2**	**3.3**	**4.3**	**5.0**	**3.2**
		CDDP	**3.3**	**4.5**	**3.7**	**3.0**	**1.7**
**IGROV-1**	Nut-3+CDDP	Nut-3	**2.3**	**3.2**	**2.5**	**1.6**	**1.2**
		CDDP	**2.9**	**2.8**	**1.8**	**1.1**	0.7
	RG+CDDP	RG7388	**1.8**	**2.5**	**5.5**	**6.2**	**4.5**
		CDDP	**3.6**	**2.4**	**1.8**	**1.1**	0.6
**OAW42**	Nut-3+CDDP	Nut-3	**1.5**	**1.6**	**1.4**	**1.1**	**1.5**
		CDDP	**3.6**	**2.9**	**2.0**	**1.4**	**1.3**
	RG+CDDP	RG7388	**2.2**	**2.1**	**1.6**	**1.1**	0.7
		CDDP	**4.5**	**3.2**	**2.0**	**1.2**	0.7

The combined treatment was performed at the indicated fixed 1:1 ratios relative to their respective GI50 concentrations. DRI values were calculated for each constant ratio combination from the average of at least three independent experiments. Favorable DRI values are highlighted in bold font. Nut-3, Nutlin-3; RG, RG7388; CDDP, cisplatin; DRI, Dose Reduction Index.

### The effect of combination treatment with Nutlin-3/RG7388 and cisplatin on p53 activation

**Figure 3:**
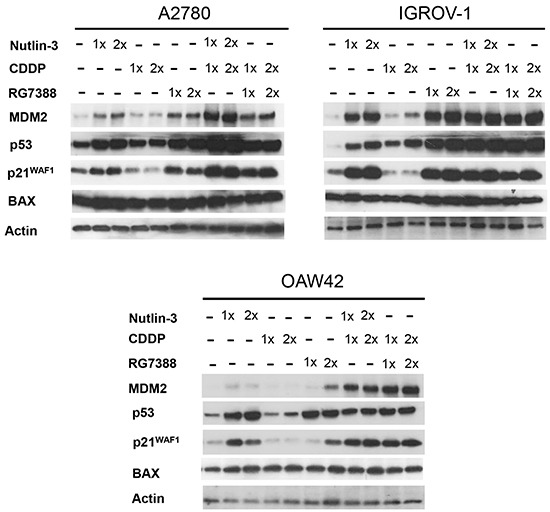
Combination of Nutlin-3/RG7388 with cisplatin increased stabilization of p53 and upregulation of its downstream targets, MDM2 and p21^WAF1^ compared to cisplatin on its own Total levels of p53, p21^WAF1^, MDM2 (4 hours) and BAX (8 hours) after the commencement of treatment with Nutlin-3 and RG7388 alone, and in combination with cisplatin at constant 1:1 ratios of 1X and 2X their respective GI_50_ concentration analyzed by western blot in three wild-type *TP53* ovarian cancer cell lines. Nutlin-3 and RG7388 led to little change in BAX compared to DMSO control and combination of Nutlin-3 with cisplatin at 1X GI_50_ showed only a slight increase in the expression of BAX in A2780 and IGROV-1 cell lines compared to cisplatin on its own.

Further analysis was performed to investigate the effect of combination treatment on the p53 molecular pathway using Western blotting. Wild-type *TP53* cell lines were treated with Nutlin-3/RG7388 and cisplatin alone, and in combination at constant 1:1 ratios of 1x and 2x their respective GI_50_ concentration for 4 hours. Western analysis showed that treatment with Nutlin-3/RG7388 and cisplatin as a single agent and in combination induced p53 stabilization and upregulation of p21^WAF1^ and MDM2, confirming functional activation of wild-type p53 (Figure 3). Moreover, combination treatment in all cases led to greater levels of p53 stabilization, as well as p21^WAF1^ and MDM2 upregulation compared to cisplatin on its own, and in most cases these were greater than those induced by Nutlin-3/RG7388 alone. Higher expression of p21^WAF1^ for combined treatment was associated with a greater synergistic effect for growth inhibition. However, Nutlin-3 and RG7388 led to little change in BAX expression compared to DMSO control, and their combination with cisplatin showed only a small increase in the expression of BAX compared to cisplatin on its own (Figure 3). Interestingly, cisplatin alone at a GI_50_ or 2x GI_50_ dose showed much less p53 pathway induction than Nutlin-3 or RG7388 alone.

### Nutlin-3/RG7388 in combination with cisplatin induces increased cell cycle distribution changes and/or apoptosis in wild-type *TP53* ovarian cancer cell lines

Wild-type *TP53* cell lines were treated with Nutlin-3/RG7388 and cisplatin, alone and in combination at constant 1:1 ratios of 1x and 2x (1/2 x & 1x for OAW42) their respective GI_50_ concentration for 24, 48 and 72 hours. They were then analyzed by flow cytometry for cell cycle phase distribution changes and evidence of apoptosis in response to treatment. The 48 and 72 hour treatment data is presented in the Supplementary Figures (S1–S4).

**Figure 4:**
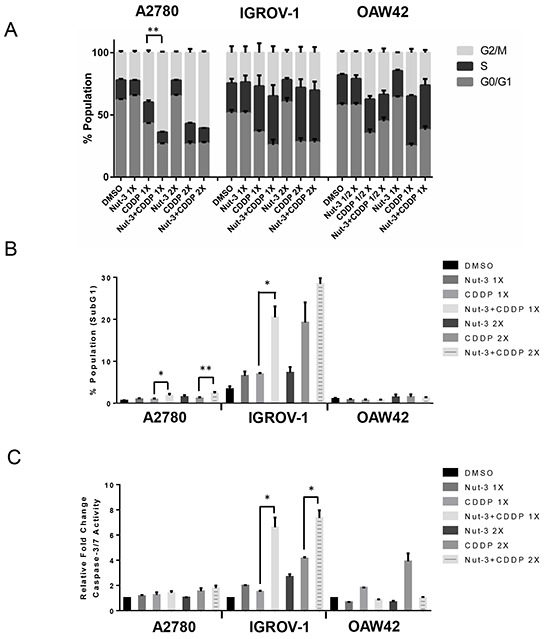
Combination of Nutlin-3 with cisplatin affects the cell cycle distribution and apoptotic endpoints Wild-type *TP53* ovarian cancer cells were treated for 24 hours with Nutlin-3 or cisplatin alone and at constant 1:1 combination ratios of 1X and 2X (1/2 X & 1X for OAW42) their respective GI_50_ concentrations. **A.** Combination of Nutlin-3 with cisplatin led to an increased proportion of cells in G2/M phase compared to either agent alone in A2780 and IGROV-1 cell lines, **B.** FACS analysis for Sub-G1 events and **C.** Caspase 3/7 activity as an indicator of apoptosis. Caspase 3/7 activity is represented as fold change relative to DMSO solvent control. Nut-3, Nutlin-3; CDDP, cisplatin; *, p < 0.05; **, P < 0.01. Data are shown as the average of at least 3 independent experiments and error bars represent SEM. Statistically significant results were only shown in comparison with cisplatin on its own.

For the wild-type *TP53* cell lines, Nutlin-3 only showed a modest increase in the proportion of cells in G0/G1 phase in a dose and time dependent manner. Nutlin-3 also increased the percentage of SubG1 events, a surrogate marker of apoptosis, in A2780 and IGROV-1 cell lines in a treatment time and dose-dependent manner (Figure 4A & 4B, Supplementary Figure S1B and Supplementary Figure S2B). For A2780 and IGROV-1 cells, combination treatment of Nutlin-3 with cisplatin led to a dose and time dependent increase in the proportion of cells in G2/M phase and the proportion of SubG1 events compared to cisplatin on its own, particularly for A2780 cells (Figure 4A & 4B, Supplementary Figure S1A & S1B and Supplementary Figure S2A & S2B).

**Figure 5:**
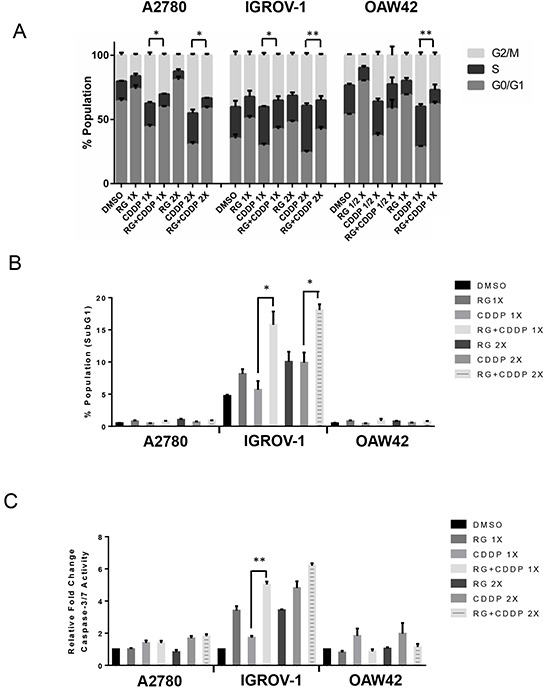
Combination of RG7388 with cisplatin affects the cell cycle distribution and apoptotic endpoints Wild-type *TP53* ovarian cancer cells were treated for 24 hours with RG7388 or cisplatin alone and at constant 1:1 combination ratios of 1X and 2X (1/2 X & 1X for OAW42) their respective GI_50_ concentrations. **A.** Combination of RG7388 with cisplatin led to an increased proportion of cells in G0/G1 phase compared to cisplatin on its own, **B.** FACS analysis for Sub-G1 events and **C.** Caspase 3/7 activity as an indicator of apoptosis. Caspase 3/7 activity is represented as fold change relative to DMSO solvent control. RG, RG7388; CDDP, cisplatin; *, p < 0.05; **, P < 0.01. Data are shown as the average of at least 3 independent experiments and error bars represent SEM. Statistically significant results were only shown in comparison with cisplatin on its own.

RG7388 alone after 24 hours treatment led to a higher increase in the proportion of cells in the G0/G1 phase of the cell cycle across all cell lines compared to Nutlin-3 at the same GI_50_ doses (Figure 5A). RG7388 induced SubG1 events in all cases in a concentration and time-dependent manner (Figure 5B, Supplementary Figure S3B and Supplementary Figure S4B). The IGROV-1 cell line showed a higher basal level of sub-G1 events on FACS analysis compared to the other cell lines, which was further increased by MDM2 inhibitor or cisplatin treatment.

In terms of the proportional distribution of cells in G0/G1 or G2/M, the effect of RG7388 combination with cisplatin was time dependent. Combined treatment for 24 hours led to proportionally more cells in the G0/G1 cell cycle phase compared to the effect of cisplatin on its own and a higher proportion of cells in the G2/M phase compared to the effect of RG7388 alone across all 3 cell lines (Figure 5A). After 48 and 72 hours treatment, the combination of RG7388 with cisplatin led to a greater proportional increase in G2/M phase compared to either agent alone for A2780 and IGROV-1, whereas for OAW42 there was a reduction in the proportion of cells in G2/M phase (Supplementary Figure S3A and Supplementary Figure S4A).

The induction of apoptosis was also evaluated by caspase 3/7 enzymatic assay, which is a sensitive and specific indicator of apoptosis [17]. Wild-type *TP53* ovarian cancer cell lines were treated for 24 hours with 1x and 2x their respective Nutlin-3/RG7388 GI_50_ concentrations as a single agent and in combination with cisplatin (Figure 4C & 5C). In general across the cell lines there was a positive correlation between the caspase 3/7 activity and accumulation of SubG1 events. With IGROV-1, a concentration-dependent increase in caspase 3/7 activity in response to Nutlin-3/RG7388 compared to DMSO control was observed. Furthermore, the combination of Nutlin-3/RG7388 with cisplatin led to more caspase 3/7 activity in IGROV-1 compared to either agent alone. For A2780 there was no significant increase in the caspase 3/7 activity in response to Nutlin-3/RG7388 alone. Also no significant increase was observed for the combination of Nutlin-3/RG7388 with cisplatin compared with the effect of cisplatin alone in the A2780 cells. Combination treatments led to a decrease in the caspase 3/7 activity in the OAW42 cells indicating a protective effect of Nutlin-3/RG7388 against cisplatin in this cell line (Figure 4C and Figure 5C). Taken together, these results demonstrated that the effect of Nutlin-3/RG7388 as a single agent and in combination with cisplatin on the cell cycle distribution, Sub-G1 events and caspase 3/7 activity is cell type, compound and time dependent.

### Nutlin-3/RG7388 alone results in clonogenic cell death in a p53-dependent manner

**Figure 6:**
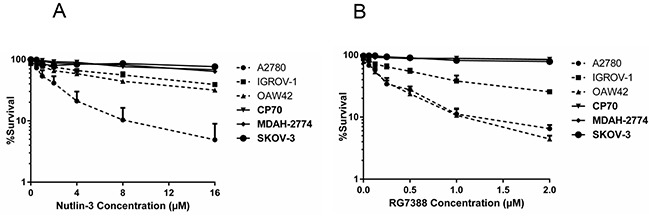
Clonogenic survival for the panel of ovarian cancer cell lines Treatment with **A.** Nutlin-3 and **B.** RG7388. Clonogenic cell survival LC_50_ values were dependent on the *TP53* genomic status. *TP53* mutant cell lines are indicated in bold font. Nut-3, Nutlin-3; RG, RG7388; CDDP, cisplatin. Data are shown as the average of at least 3 independent experiments and error bars represent SEM.

Clonogenic survival assays were performed for the panel of six ovarian cancer cell lines. Exponentially proliferating cell cultures were counted and seeded at appropriate densities for colony formation and treated with different concentrations of Nutlin-3/RG7388. The results showed *TP53* mutant cell lines were significantly more resistant to Nutlin-3/ RG7388, but also demonstrated a wide range of responses for the wild-type *TP53* cell lines (Figure 6A & Table 4). Nutlin-3 markedly decreased the clonogenic survival of A2780 cells (LC_50_= 1.65 ± 0.7 (SEM) μM); however, IGROV-1 (LC_50_= 11 ± 2.1(SEM) μM) and OAW42 (LC_50_= 6.25 ± 0.50 (SEM) μM) were substantially less sensitive to Nutlin-3 (Figure 6A).

**Table 4:** LC_50_ concentrations for cisplatin, Nutlin-3 and RG7388 for the panel of ovarian cancer cell lines of varying *TP53* status

Cell line	*TP53* status	Cisplatin (μM)	Nutlin-3 (μM)	RG7388 (μM)
**A2780**	Wild-type	0.42 ± 0.0003	1.65 ± 0.71	0.14 ± 0.03
**IGROV-1**	Wild-type	0.82 ± 0.06	11 ± 2.08	0.67 ± 0.15
**OAW42**	Wild-type	0.28 ± 0.01	6.25 ± 0.50	0.15 ± 0.04
**CP70**	Mutant	3.39 ± 0.05	> 32	>2
**MDAH-2774**	Mutant	0.67 ± 0.08	> 32	>2
**SKOV-3**	Mutant	1.87 ± 0.42	> 32	>2

Data represent the mean of at least 3 independent experiments ± SEM.

RG7388 was much more potent than Nutlin-3, and decreased the clonogenic survival of all the wild-type *TP53* ovarian cancer cell lines. Consistent with the mechanism of action for MDM2 antagonists, RG7388 had little or no effect on mutant *TP53* cell lines in the 0-2μM dose range (Figure 6B). Interestingly, although all three cell lines were sensitive to RG7388, the relative sensitivity of the wild-type *TP53* cell lines to Nutlin-3 and RG7388 was very different. The clonogenic cell survival response to RG7388 for A2780 and OAW42 was similar, whereas for Nutlin-3 their relative responses were quite different, with only A2780 showing sensitivity to Nutlin-3. Overall, the clonogenic cell survival assays showed not only that mutant *TP53* genomic status was a major determinant of resistance to Nutlin-3 and RG7388, but also that the relative response of the wild-type *TP53* cell lines differed for the two MDM2 inhibitors in a way that was not explicable simply by the relative potency of the compounds in cell-free MDM2-p53 binding assays.

### Nutlin-3/RG7388 and cisplatin are synergistic for clonogenic cell killing of wild-type *TP53* ovarian cancer cell lines

**Figure 7:**
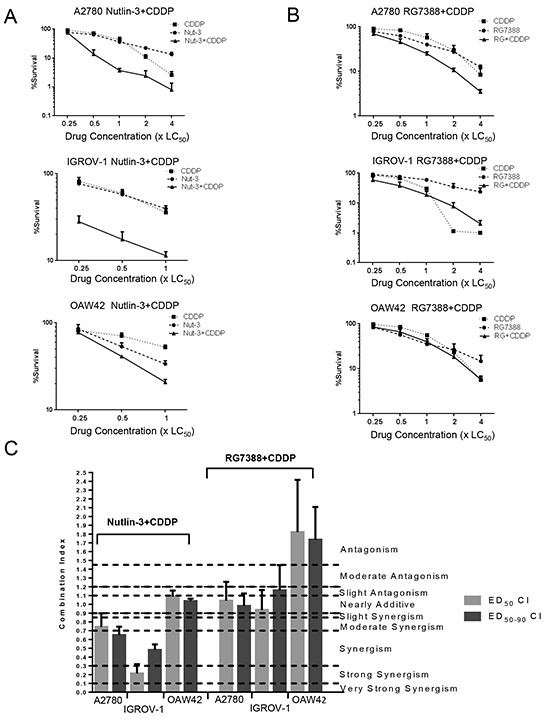
Nutlin-3/RG7388 has a synergistic or additive effect with cisplatin in clonogenic survival assays in wild-type *TP53* ovarian cancer cells **A.** Clonogenic survival for three wild-type *TP53* cell lines exposed to Nutlin-3 and CDDP alone, and in combination at constant 1:1 ratios of 0.25X, 0.5X, 1X, 2X and 4X (A2780) and 0.25X, 0.5X, 1X (IGROV-1 & OAW42) their respective LC_50_ concentration for 48 hours. **B.** Clonogenic survival for three wild-type *TP53* cell lines exposed to RG7388 and CDDP alone, and in combination at constant 1:1 ratios of 0.25X, 0.5X, 1X, 2X and 4X their respective LC_50_ concentration for 48 hours. **C.** The CI values for Nutlin-3/RG7388 in combination with cisplatin at ED_50_ and, the average of CI values at effect levels ED_50_, ED_75_ and ED_90_ in three wild-type *TP53* ovarian cancer cell lines. Data are shown as the average of at least 3 independent experiments and error bars represent SEM. Nut-3, Nutlin-3; RG, RG7388; CDDP, cisplatin, CI, Combination Index.

The reduction in clonogenic survival in response to 48 hours exposure to Nutlin-3, RG7388 and cisplatin, both as single agents and in combination at five equipotent concentrations between 0.25× and 4× their respective LC_50_ concentrations was determined for the three wild-type *TP53* cell lines and evaluated by median effect analysis. Due to the high LC_50_ for IGROV-1 and OAW42 in response to Nutlin-3, 3 equipotent concentrations between 0.25× and 1x their respective LC_50_ concentrations were used to assess the combination effect of Nutlin-3 with cisplatin. The effect of combined treatment was cell type and compound dependent (Figure 7A & 7B). The combination of Nutlin-3 with cisplatin led to a further decrease in colony formation compared to treatment with either agent alone for all three cell lines and was particularly marked for IGROV1.

Although the combination of Nutlin-3 with cisplatin significantly decreased the clonogenic survival of IGROV-1 and A2780 compared to either agent alone, the combined treatment of RG7388 with cisplatin at the same LC_50_ ratios only moderately reduced colony formation (Figure 7A & 7B). For the OAW42 cell line, the combination treatment of Nutlin-3 with cisplatin reduced the ability of the OAW42 cell line to form colonies to a greater extent than either agent on its own. In contrast, there was no significant reduction in the clonogenic cell survival of OAW42 following combination treatment with RG7388 and cisplatin compared to either agent alone (Figure 7A & 7B).

The data were analyzed using median-effect analysis and CI values calculated to evaluate whether the observed differences in clonogenic cell survival were synergistic, additive or antagonistic. CI values for each constant ratio combination at estimated effect levels of ED_50_, ED_75_ and ED_90_ were individually computed, and the average of CI values was also determined. Across all three wild-type *TP53* cell lines, the effect of combination treatment of Nutlin-3 with cisplatin ranged from additive to strongly synergistic (Table 5 and Figure 7C). In addition, for combination treatments, both Nutlin-3 and cisplatin had a favorable DRI ranging from 1.3-fold to 10.9-fold dose reduction (Table 6).

**Table 5:** Clonogenic survival CI values for RG7388/Nutlin-3 in combination with cisplatin for the wild-type *TP53* ovarian cancer cell lines

Cell Line	Combination	CI					CI ED_50_	CI ED_75_	CI ED_90_	CI Ave ED_50-90_
		XGI50								
		0.25	0.5	1	2	4				
**A2780**	Nut-3+CDDP	1.9	**0.4**	**0.4**	**0.6**	**0.6**	**0.7**	**0.6**	**0.6**	**0.7**
	RG+CDDP	1.2	1.0	1.0	1.0	**0.9**	1.0	1.0	**0.9**	1.0
**IGROV-1**	Nut-3+CDDP	**0.4**	**0.5**	**0.7**	ND	ND	**0.2**	**0.4**	**0.9**	**0.5**
	RG+CDDP	**0.8**	1.0	1.4	1.9	1.9	**0.9**	1.1	1.4	1.1
**OAW42**	Nut-3+CDDP	1.8	1.0	1.0	ND	ND	1.1	1.0	1.0	1.0
	RG+CDDP	1.2	1.9	1.7	1.7	1.7	1.8	1.7	1.7	1.7

The combined treatment was performed at the indicated fixed 1:1 ratios relative to their respective GI50 concentrations. CI values were calculated for each constant ratio combination and at effect levels ED50, ED75 and ED90 from the average of at least three independent experiments. CI Ave ED50-90 represents the average of CI values at effect levels of ED50, ED75 and ED90. CI range: < 0.1 very strong synergism; 0.1-0.3 strong synergism; 0.3-0.7 synergism; 0.7-0.85 moderate synergism; 0.85-0.9 slight synergism; 0.9-1.1 nearly additive; 1.1-1.2 slight antagonism; 1.2-1.45 moderate antagonism; 1.45-3.3 antagonism; 3.3-10 strong antagonism; > 10 very strong antagonism. Synergistic combinations are highlighted in bold font. Nut-3, Nutlin-3; RG, RG7388; CDDP, cisplatin; CI, Combination Index; ND; Not determined.

**Table 6:** DRI values for clonogenic cell killing by RG7388/Nutlin-3 in combination with cisplatin in wild-type *TP53* ovarian cancer cell lines

Cell Line	Combination	Component	DRI				
			XGI50				
			0.25	0.5	1	2	4
**A2780**	Nut-3+CDDP	Nut-3	**1.3**	**7.3**	**10.7**	**7.8**	**10.9**
		CDDP	**2.1**	**4.0**	**3.4**	**2.2**	**2.0**
	RG+CDDP	RG7388	**1.6**	**1.9**	**2.0**	**2.5**	**3.5**
		CDDP	**3.2**	**2.6**	**2.1**	**1.8**	**1.7**
**IGROV-1**	Nut-3+CDDP	Nut-3	**6.4**	**5.4**	**4.1**	ND	ND
		CDDP	**5.1**	**3.7**	**2.2**	ND	ND
	RG+CDDP	RG7388	**4.3**	**4.3**	**4.3**	**4.5**	**11.0**
		CDDP	**2.1**	**1.4**	0.9	0.6	0.6
**OAW42**	Nut-3+CDDP	Nut-3	**1.6**	**1.5**	**1.3**	ND	ND
		CDDP	**1.8**	**3.2**	**5.0**	ND	ND
	RG+CDDP	RG7388	1.0	**1.1**	**1.2**	**1.4**	**2.0**
		CDDP	**2.1**	**1.7**	**1.4**	**1.1**	0.9

The combined treatment was performed at the indicated fixed 1:1 ratios relative to their respective LC50 concentrations. DRI values were calculated for each constant ratio combination from the average of at least three independent experiments. Nut-3, Nutlin-3; RG, RG7388; CDDP, cisplatin; DRI, Dose Reduction Index; ND; Not determined; Favorable DRI values are highlighted in bold font.

For A2780 and IGROV-1 cell lines a synergistic effect was observed for combination treatment with Nutlin-3 and cisplatin, whereas for RG7388 and cisplatin combinations the effect was additive to antagonistic. For the OAW42 cell line the combination of RG7388 and cisplatin was antagonistic, suggesting RG7388 had a protective effect against cisplatin (Table 5 and Figure 7C). Although the combined effect of RG7388 with cisplatin ranged from additive to antagonistic, there was nevertheless a favorable DRI for the same level of clonogenic cell killing when treatments are combined for all RG7388 concentrations and most concentrations of cisplatin (Table 6).

### Nutlin-3/RG7388 induces expression of cell cycle arrest/ apoptosis-related genes and those involved in response to DNA repair

**Figure 8:**
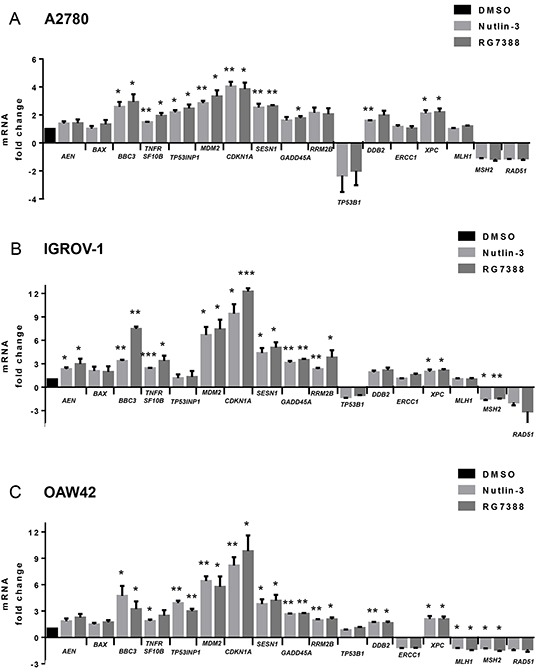
mRNA expression of genes relating to apoptosis, cell cycle arrest, nucleotide excision repair (NER) and DNA mismatch repair in response 5 μM Nutlin-3 or 0.5 μM RG7388 for 6 hours relative to DMSO solvent control **A.** A2780, **B.** IGROV-1 and **C.** OAW42, *, p < 0.05; **, P < 0.01. Data are presented as mean ± standard error of mean (SEM) of three independent repeats.

**Figure 9:**
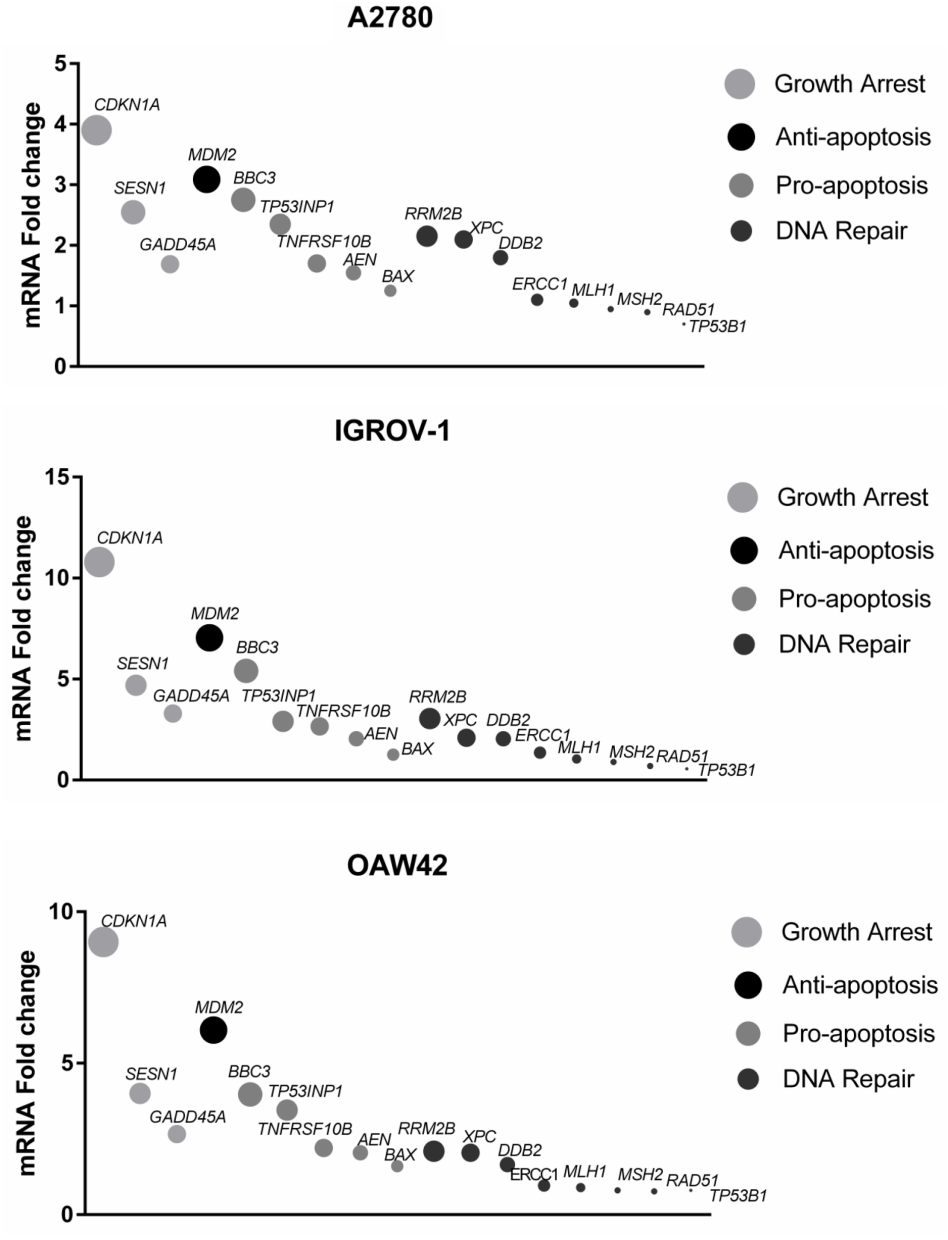
Growth arrest, pro-apoptotic, anti-apoptotic and DNA repair-related gene expression changes induced by 5 μM Nutlin-3 or 0.5 μM RG7388 for 6 hours relative to DMSO solvent control Summary data are presented as a combination of three independent repeats for Nutlin-3 and three for RG7388.

To investigate the mechanistic basis for the observed combination effects, the effect of MDM2 inhibitor treatment on mRNA expression of candidate genes with potential for influencing the response to cisplatin was analyzed by quantitative real time polymerase chain reaction (qRT-PCR). Changes in the expression of cell cycle/apoptosis-related genes as well as those involved in nucleotide excision repair (NER) and mismatch repair (MMR) for the three wild-type *TP53* cell lines in response to Nutlin-3 and RG7388 are shown in Figure 8 & 9. The cells were treated with 5 (μM) Nutlin-3 and 0.5 (μM) RG7388, and the total RNA was extracted 6 hours after the commencement of treatment.

Overall, the fold changes in expression in response to MDM2 inhibitors were less in A2780 cells than IGROV-1 and OAW42 (Figure 8 & 9). In the case of the genes involved in cell cycle arrest and growth inhibition, Nutlin-3 and RG7388 treatment significantly induced *CDKN1A*, *SESN1* and *GADD45A* gene expression in all three cell lines, with *CDKN1A* consistently showing the highest level of induction (*p<0.05*) (Figure 8 & 9). Both treatments showed a significant increase in the expression of the pro-apoptotic *TNFRSF10B* and *BBC3* (*PUMA)* genes in all three cell lines, with increases of *BBC3* mRNA being highest in the IGROV-1 cell line (*p<0.05*) (Figure 8). The Nutlin-3/RG7388 treatment also increased expression of the pro-apoptotic gene *TP53INP1* in A2780 and OAW42 cells; however, there was no significant induction in IGROV-1. No significant increase was observed for the pro-apoptotic gene *BAX* in any of the cell lines. Although Nutlin-3/RG7388 treatment led to significantly increased expression of *AEN* in IGROV-1, the induction of *AEN* was not statistically significant for A2780 and OAW42 (Figure 8). Furthermore, both Nutlin-3 and RG7388 treatments significantly increased the expression of the anti-apoptotic *MDM2* gene across all three cell lines. The treatments led to a statistically significant decrease in the expression of BCL-2 although the changes were small and unlikely to be biologically significant. Also no significant changes were observed in the expression levels of the anti-apoptotic *BIRC5* and *MCL-1* genes (Supplementary Figure S5).

To study the effect of Nutlin-3 and RG7388 on the expression of genes implicated in the response to DNA repair induced by cisplatin, the expression of *TP53BP1*, *DDB2*, *ERCC1*, *XPC*, *MLH1*, *MSH2*, *RAD51* and *RRM2B* genes in response to Nutlin-3 and RG7388 was investigated. A significant increase was measured in the expression of *DDB2* in response to Nutlin-3 and *XPC* in response to both Nutlin-3 and RG7388 for A2780 cells *(p<0.05*). In the case of IGROV-1, *XPC* and *MSH2* gene expression levels were significantly induced and reduced respectively in response to both Nutlin-3 and RG7388 (*p<0.05*). For OAW42 cells there was a significant increase in the expression of *DDB2* and *XPC* genes and a significant decrease in the *MLH1* and *MSH2* expression levels in response to Nutlin-3 and RG7388 treatment (*p*<0.05). With Nutlin-3 treatment, the *TP53BP1* gene expression decreased in all three cell lines, although statistically this trend was not significant (*p>0.05*).

The mRNA profile found for *CDKN1A* and *BAX* genes was consistent with the western blot analysis for p21^WAF1^ and BAX proteins (Figure 1B, 1C and Figure 3). The increased *CDKN1A* and *SESN1* gene expression is also in agreement with induced cell cycle arrest and growth inhibition across the three cell lines. Furthermore, induction of *BBC3* and *TNFRSF10B* is in accordance with the induction of apoptosis, Sub-G1 events and caspase 3/7 activity, in A2780 and IGROV-1 (Figure 4B, Figure 4C, Figure 5B and 5C). However, in spite of significantly increased *BBC3* and *TNFRSF10B* gene expression levels in OAW42, no induction of apoptosis was observed in this cell line (Figure 4B, Figure 4C, Figure 5B and 5C).

The increased sensitivity to MDM2 inhibitors and their synergy with cisplatin observed with the A2780 cells was not obviously attributable to any individual change in candidate gene expression. However, as can be seen in Figure 9, the balance of expression between the growth arrest genes and the autoregulatory negative feedback *MDM2* gene on the one hand and the pro-apoptotic and DNA repair genes on the other hand, was clearly less with the A2780 cells.

## DISCUSSION

Advanced ovarian cancer treatment usually involves a combination of debulking surgery and platinum based chemotherapy, alone or with the addition of paclitaxel. Although chemotherapy prolongs survival, most patients with advanced disease die from treatment resistant progressive disease [18]. Cancer therapy has recently been improving with the introduction of targeted therapies to achieve greater specificity and less cytotoxicity [19]. This study evaluates for the first time the effect of the MDM2-p53 binding antagonist RG7388, as a single agent in a panel of ovarian cancer cell lines of defined *TP53* genomic status. It also investigates the effect of RG7388 or Nutlin-3 in combination with cisplatin in wild-type *TP53* ovarian cancer cell lines and explores the mechanistic basis for the growth inhibitory and cytotoxic responses.

Within the panel of ovarian cancer cell lines studied, wild-type *TP53* cell lines were significantly more sensitive to Nutlin-3 and the more potent RG7388 compared to mutant *TP53* cell lines. Consistent with their mechanism of action, Nutlin-3 and RG7388 treatment led to more p53 stabilization and induction of p21^WAF1^ and MDM2 in the wild-type *TP53* ovarian cancer cell lines. There was no upregulation of p53 downstream target genes in *TP53* mutant ovarian cancer cell lines in response to Nutlin-3 and RG7388 treatment [20]. Interestingly, despite the lack of downstream function, there was some evidence of mutant p53 stabilization in response to treatment with MDM2 inhibitors in the *TP53* mutant CP70 and *MLH1*-corrected CP70+ cell lines. This suggests some mutant forms of p53 still show evidence of degradation by MDM2 which is prevented by the MDM2 inhibitors. These results are consistent with limited previous studies [10, 21] demonstrating that wild-type *TP53* ovarian cancer cell lines are responsive to Nutlin-3 and extends observations to the second generation MDM2 inhibitor RG7388 currently in early phase clinical trials.

Resistance to MDM2-p53 binding antagonists has been suggested to be acquired by prolonged exposure of cells to sub-lethal doses through *de novo* inactivating *TP53* mutations or selection of pre-existing subclones of *TP53* mutant cells that might be present as a result of cancer cell genomic instability and tumor heterogeneity [22, 23]. For this reason, it is suggested that MDM2-antagonists are likely be most effective in combination with standard existing chemotherapeutic agents or agents that target or limit the potential outgrowth of *TP53* mutated cells. The major benefits of combination therapy are the potential for a synergistic therapeutic effect, dose and toxicity reduction, and delay or prevention of drug resistance [24, 25]. Platinum agents used to treat ovarian cancer have major adverse side effects including nephrotoxicity, ototoxicity, myelosuppression and gastrointestinal disorders [26]. This study set out with the aim of assessing the effect of single and combination treatment of Nutlin-3 and RG7388 with cisplatin in a panel of ovarian cancer cell lines of known *TP53* status. The effects observed were compound and cell type dependent. Overall, the combination effect of Nutlin-3 and RG7388 with cisplatin ranged from synergism in A2780 and moderate synergism to antagonism in IGROV-1 and OAW42 cell lines. A single limited previous study examined the combination of Nutlin-3a with cisplatin in A2780p, A2780cis and the OV90 cell lines. The results showed a synergistic effect in A2780p and A2780cis, consistent with our study [10].

Even in the absence of synergy, combined treatment can nevertheless be of potential clinical use, because in most cases a favorable dose reduction for each agent may still be achievable for a given level of effect compared with each agent alone (Tables 3 and 6). This is of particular potential benefit when the agents in question, in this case MDM2 inhibitors and cisplatin, have different dose limiting toxicities. [27].

The combination treatments increased stabilization of p53 and upregulation of p21^WAF1^ compared to either agent alone, particularly compared to cisplatin in A2780 and IGROV-1. However, there was no significant increase in p53 stabilization and p21^WAF1^ upregulation in combined treatment of OAW42 cells compared to Nutlin-3 and RG7388 as single treatments. These results help to understand the observed differences in the effect of combined treatment on growth inhibition and clonogenic cell survival between these cell lines. These findings are in keeping with functional activation of p53 as a driver of the synergistic effects in combination treatment. For growth inhibition the increased upregulation of p21^WAF1^ is consistent with its role in cell cycle arrest [28].

Individually, Nutlin-3 and RG7388 induced cell cycle arrest in wild-type *TP53* ovarian cancer cell lines in a time and dose-dependent manner. When cells were treated with the GI_50_ isoeffect doses of RG7388 or Nutlin-3, RG7388 had a greater effect on the cell cycle distribution, with increased accumulation of cells in G0/G1. Combination treatment with Nutlin-3 or RG7388 and cisplatin led to greater G2/M and/or G0/G1 cell cycle phase accumulation, more SubG1 events and/or higher levels of caspase 3/7 activity compared to either agent alone in a cell type and time dependent manner. Overall, there was a positive correlation between the detection of SubG1 events on FACS analysis and caspase 3/7 activity, however cell cycle arrest is not always accompanied by the induction of apoptosis [29] as seen for OAW42 cells in this study. The apparent protective effect of Nutlin-3/RG7388 against cisplatin in OAW42, indicated by the antagonistic effect of combination (Figure 2C and 7C), was reflected by fewer SubG1 events and Caspase 3/7 activity compared to cisplatin on its own.

The clonogenic cell survival assays also showed *TP53* mutant cell lines were much more resistant to Nutlin-3 and RG7388, but nevertheless also demonstrated a range of different relative single agent responses for the wild-type *TP53* cell lines (Figure 6 and Table 4). A possible explanation for this range of responses between the wild-type *TP53* cell lines might be due to differences in drug uptake [30] or deficiencies or variation in the expression of p53 target genes involved in apoptosis and other mechanisms of cell death, including the pro-apoptotic proteins BAX and PUMA and anti-apoptotic proteins BCL-2 and MCL-1 [31, 32].

Combined treatment with Nutlin-3 and cisplatin significantly decreased the clonogenic survival of wild-type *TP53* ovarian cancer cells compared with either agent alone, and the combination effect ranged from additive to strong synergy. Nutlin-3 may sensitize wild-type *TP53* ovarian cancer cell lines to cisplatin via multiple factors including increased p53-dependent apoptosis [33, 34]. Surprisingly, the combination of RG7388 with cisplatin showed no evidence of synergy for reduction of colony forming ability. The clonogenic assay results for combination of RG7388 with cisplatin ranged from antagonism for OAW42, indicating a protective effect of RG7388 against cisplatin, to additive for A2780 and IGROV-1. The difference between the results for combination of cisplatin with Nutlin-3 compared to the combination with RG7388 may in part be due to different p53-dependent off-target effects of these MDM2 inhibitors. A contributory factor may be differences in G1 cell cycle arrest with MDM2 inhibitors, since an increased G1 cell cycle arrest may protect against agents such as cisplatin which are preferentially cytotoxic against S-phase cells [35].

Across the 3 cell lines, Nutlin-3/RG7388 increased *CDKN1A* and *SESN1* expression consistent with their essential role in cell cycle arrest and growth inhibition [36, 37]. Both Nutlin-3 and RG7388 treatment significantly induced the expression of the pro-apoptotic *TNFRSF10B* and *BBC3* genes in all 3 cell lines. The TNFRSF10B receptor and its ligand, TRAIL, have been reported to preferentially induce apoptosis in transformed and tumor cells even though it is expressed at a significant level and may be induced in normal tissues [38, 39]. This may contribute to the generally greater toxicity of MDM2 inhibitors for cancer cells compared to normal cells, although some haematopoetic cell lineages also appear to be sensitive, as evidenced by the dose limiting thrombocytopenia seen in the early phase clinical trials of MDM2 inhibitors [40]. There was a positive concordance between the expression of these pro-apoptotic genes and the apoptotic endpoints shown by Sub-G1 signals on FACS analysis and caspase 3/7 activity in A2780 and IGROV-1 cell lines. However, this relationship did not extend to the OAW42 cell line, which despite increased *TNFRSF10B*, *TP53INP1* and the highest pro-apoptotic *BBC3* expression was not in keeping with low caspase 3/7 activity and SubG1 FACS signals. Failure to undergo apoptosis in OAW42 cells in response to C1311, a new class of imidazoacridinones, has also been reported, consistent with our observation [41].

A possible explanation for the lack of evidence of apoptosis in OAW42 might be high expression of anti-apoptotic proteins such as BCL-2, BCL-X and MCL-1, or deficiency in downstream factors involved in the apoptosis cascade [27, 28]. Although BAX is required for PUMA-induced apoptosis, there was no significant increase in *BAX* expression, either at the mRNA or protein level in response to Nutlin-3/RG7388 treatment in any of the cell lines.

Significantly increased expression of several p53-regulated genes involved in the repair of DNA lesions induced by cisplatin, including *DDB2*, *XPC* and *RRM2B*, lead us to reject the hypothesis that reduced capacity for repair of cisplatin induced DNA damage leads to a synergistic effect in combined treatment with Nutlin-3/RG7388 and cisplatin. Although there was some evidence of a reduced expression of the DNA mismatch repair genes, *MLH1* and *MSH2*, the changes were very small and unlikely to be biologically significant.

In conclusion, the present study demonstrates that RG7388 has activity as a single agent against wild-type *TP53* ovarian cancer cells, leading to cell cycle arrest and/or apoptosis. In addition, combination treatment with MDM2 inhibitors and cisplatin has synergistic and/or dose reduction potential dependent on cell genotype and compound and merits further investigation. Our study clearly indicates that the presence of wild-type *TP53* remains the main predictive biomarker of response to MDM2 inhibitors. However, additional determinants of response involve the balance of activity between growth inhibitory/pro-survival and pro-apoptotic genes and our results indicate that this dominates the small changes in the expression of DNA repair genes as an explanation for the synergy observed for treatment with cisplatin and MDM2 inhibitors.

## MATERIALS AND METHODS

### Chemicals and antibodies

Nutlin-3, a 1:1 mixture of the active enantiomer Nutlin-3a and the inactive enantiomer Nutlin-3b, was purchased from NewChem (Newcastle, UK) and RG7388 was kindly provided by Professor Herbie Newell and made available by the Newcastle Anticancer Drug Development Initiative. Both were dissolved in dimethyl sulfoxide (DMSO). Cisplatin (Merck Millipore, Watford, UK) was dissolved in distilled water.

### Cell lines

The ovarian cancer cell lines used in this study, their *TP53* status and histological subtype are listed in Table 1. All cell lines were sourced from the NICR authenticated cell bank and regularly tested for Mycoplasma. A2780, IGROV-1, OAW42 and CP70 were cultured in RPMI-1640 supplemented with 10% (v/v) FBS and 5% (v/v) penicillin/streptomycin. The CP70 cell line harbors a heterozygous *TP53* mutation (c.514 G->T, p.Val172Phe) [42]. The *MLH1*-corrected CP70+ cell line was grown in RPMI-1640 supplemented with 10% (v/v) FBS and Hygromycin B (200 μg/ml: Life Technologies, Inc.) [42]. This cell line has the heterozygous *TP53* mutation (c.514 G->T, p.Val172Phe). SKOV-3 and MDAH-2774 cell lines were cultured in DMEM supplemented with 10% and 5% (v/v) FBS and penicillin/streptomycin respectively. As information on the *TP53* status of SKOV-3 in the literature was contradictory, sequencing was performed and a frame shift deletion (c.265delC, p.Pro89fsX33) was confirmed (Supplementary Figure S5). The MDAH-2774 harbors a *TP53* mutation located in exon 8 (c.818G->A, p.Arg273His) [43].

### Growth inhibition assays and median-effect analysis

The GI_50_ values, the required concentrations of each compound leading to 50% growth inhibition, were determined by Sulforhodamine B (SRB) growth inhibition assays for drug exposure over 72 hours and the absorbance of the re-dissolved SRB protein stain was measured at 570 nm using a 96-well plate spectrophotometer (Spectramax 250 Molecular Devices) [44]. Growth curves were constructed using GraphPad Prism statistical analysis software version 5.04. For combination treatment of Nutlin-3 or RG7388 with cisplatin, the wild-type *TP53* cell lines were treated for 72 hours with each agent alone and in combination simultaneously at constant 1:1 ratios of 0.25x, 0.5x, 1x, 2x, and 4x their respective GI_50_ concentrations. Median-effect analysis was used to calculate Combination Index (CI) and Dose Reduction Index (DRI) values [27] using CalcuSyn software v2 (Biosoft, Cambridge, UK).

### Western blotting

Lysis buffer (12.5 ml Tris HCL, 2g SDS, 10 ml Glycerol, 67.5 ml Distilled Water) was used to harvest whole-cell lysates, followed by sonication. The concentration of protein in the cell lysates was estimated by using a bicinchoninic acid (BCA) assay. Novex® 4-20% Tris-Glycine 12-well polyacrylamide gradient gels (Invitrogen, UK) were used to separate proteins. The separated proteins were transferred by perpendicular electrophoresis to a nitrocellulose Hybond^TM^ C membrane (Amersham, Buckinghamshire, UK). Monoclonal Mouse Anti-Human primary antibodies Actin 1:1000 (#: A4700, Sigma-Aldrich), MDM2 1:300 (#: OP46-100UG, Merck Millipore), p21^WAF1^ 1:100 (#: OP64, Calbiochem), p53 1:500 (#: NCL-L-p53-DO7, Leica Microsystems Ltd.) and Polyclonal Rabbit Anti-Human primary antibody BAX 1:1000 (#: 2772S, Cell Signalling) were used. Secondary goat anti-mouse/rabbit HRP-conjugated antibodies (#: P0447/P0448, Dako) were used at 1:1000. All antibodies were diluted in 5% milk/1XTBS-Tween (w/v). Enhanced chemiluminescence (GE Life Sciences) and X-ray film (Fujifilm) were used to visualize the proteins.

### Flow cytometry

Cells were treated with Nutlin-3, RG7388 and cisplatin alone and with combinations of Nutlin-3 or RG7388 with cisplatin simultaneously at constant 1:1 ratios of 1x and 2x (A2780 and IGROV-1) or 0.5x and 1x (OAW42) their respective GI_50_ concentrations for 24, 48 and 72 hours. Cells harvested by trypsinisation were washed with PBS and resuspended in 500 μL PBS with 1mg/mL sodium citrate (Sigma, St Louis, MO), 100 μg/mL propidium iodide (Sigma), 200 μg/mL RNAse A (Sigma) and 0.3% Triton-X (Sigma). Samples were analyzed on a FACSCalibur^TM^ flow cytometer using CellQuest Pro software (Becton Dickinson, Oxford, UK). Cell cycle distribution was determined using Cyflogic (CyFlo Ltd, Turku, Finland).

### Clonogenic cell survival assay

Based on the drug concentration and sensitivity of cell lines, 100 to 100,000 cells were seeded into triplicate six-well plates. Cells were treated with Nutlin-3, RG7388 and cisplatin alone and in combination at constant 1:1 ratios of 0.25x, 0.5x, 1x, 2x and 4x or 0.25x, 0.5x and 1x their respective LC_50_ concentrations, depending on the cell line and its single agent LC_50_ values, for 48 hours. Then, the medium including drug was removed and drug-free medium was added. The cells were incubated for 2 to 3 weeks to form colonies for counting (a colony was defined as a focus of ≥50 cells). After that, the plates were washed with PBS, fixed with methanol/acetic acid (3:1) and stained with 0.5% (w/v) crystal violet. Plates were again washed and then left at room temperature to dry. The LC_50_ values were calculated using GraphPad Prism statistical analysis software version 5.04. DRI and CI values were calculated by median-effect analysis [27] using CalcuSyn software v2 (Biosoft, Cambridge, UK).

### Caspase 3/7 activity assay

Caspase 3/7 activity was measured using a Caspase- Glo 3/7 assay following the manufacturer's instructions (Promega, Southampton, UK).

### qRT-PCR

Total RNA was extracted using an RNeasy Mini Kit (Qiagen, Germany). RNA purity and concentration were estimated with an ND-1000 spectrophotometer (NanoDrop Technologies, Thermo Scientific, UK). Total messenger RNA was converted to cDNA using the Promega Reverse Transcription System (A3500, Promega) as described by the manufacturer. Validated primers used (Sigma-Aldrich UK) are listed in Supplemental Table 1 & 2. qRT-PCR was carried out using SYBR® green RT-PCR master mix (Life technologies) according to the manufacturer's guidelines. PCR reactions with 50ng/μl of the cDNA samples per 10μl final reaction volume, were performed using standard cycling parameters (Stage 1: 50°C for 2min, Stage 2: 95°C for 10min then 40 cycles of 95°C for 15 Sec and 60°C for 1 min) on an ABI 7900HT sequence detection system. *GAPDH* was used as endogenous control and the DMSO solvent control sample used as the calibrator for each independent repeat. Data analysis using the ΔΔCt Method was carried out using SDS 2.2 software (Applied Biosystems).

### Statistical analysis

All statistical tests presented were carried out using GraphPad Prism version 5.04 software. A p-value of <0.05 was considered to be statistically significant based on at least n=3 experimental repeats.

## SUPPLEMENTARY FIGURES AND TABLES


