# Supplementary material for: Pre-clinical efficacy and synergistic potential of the MDM2-p53 antagonists, Nutlin-3 and RG7388, as single agents and in combined treatment with cisplatin in ovarian cancer
Source: Oncotarget. 2016 May 20;7(26):40115–34. doi: 10.18632/oncotarget.9499 (PMC5129997; doi:10.18632/oncotarget.9499)
Supplement: Supplementary file 1 [file oncotarget-07-40115-s001.pdf]

Pre-clinical efficacy and synergistic potential of the MDM2-p53 antagonists, Nutlin-3 and RG7388, as single agents and in combined treatment with cisplatin in ovarian cancer

SUPPLEMENTARY FIGURES AND TABLES

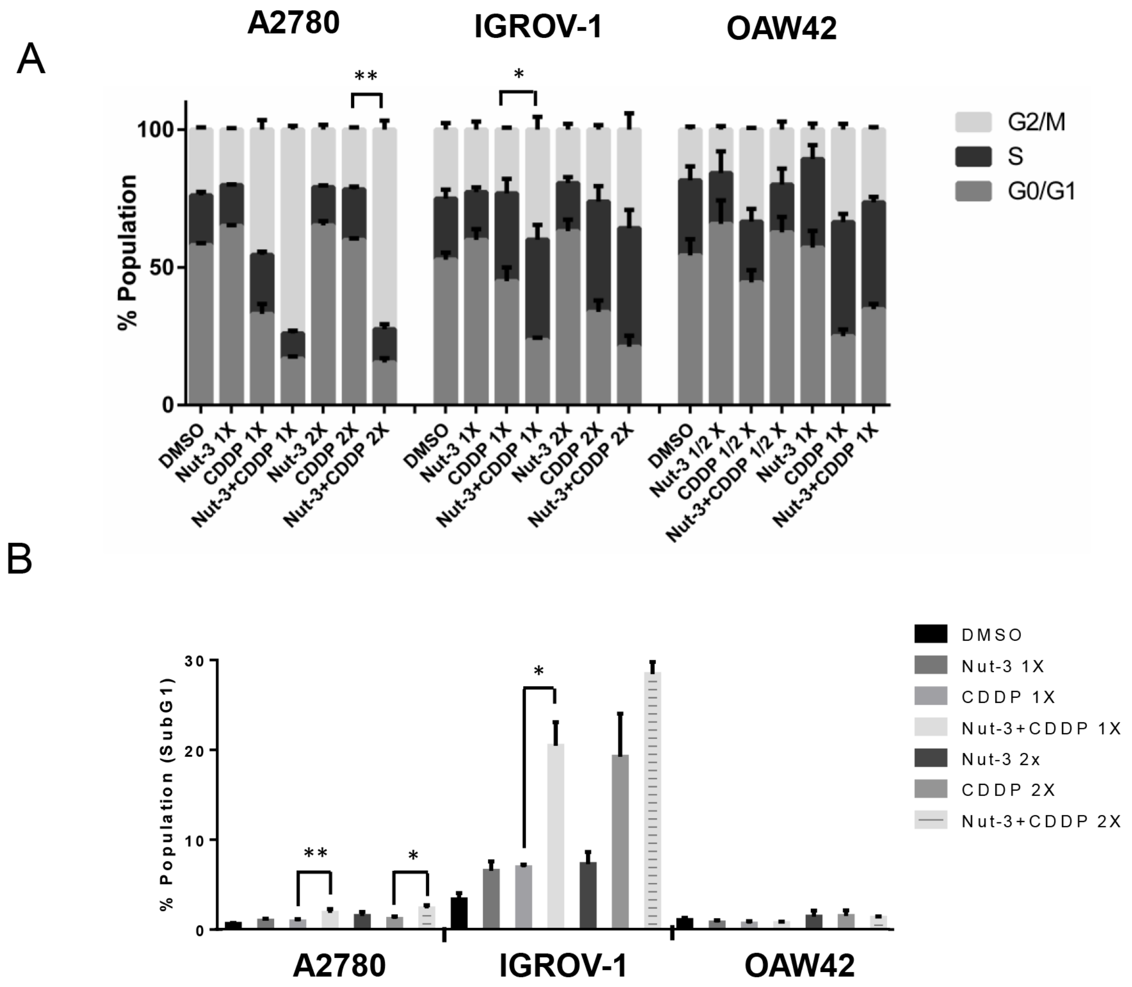

Supplementary Figure S1: Combination of Nutlin-3 with cisplatin affects the cell cycle distribution and apoptotic endpoints. Wild-type *TP53* ovarian cancer cells were treated for 48 hours with Nutlin-3 or cisplatin alone and at constant 1:1 combination ratios of 1X and 2X (1/2 X & 1X for OAW42) their respective GI_{50} concentrations. **A.** Combination of Nutlin-3 with cisplatin led to an increased proportion of cells in G2/M phase compared to either agent alone in A2780 and IGROV-1 cell lines and **B.** FACS analysis for Sub-G1 events. Nut-3, Nutlin-3; CDDP, cisplatin; *, $p < 0.05$; **, $P < 0.01$. Data are shown as the average of at least 3 independent experiments and error bars represent SEM. Statistically significant results were only shown in comparison with cisplatin on its own.

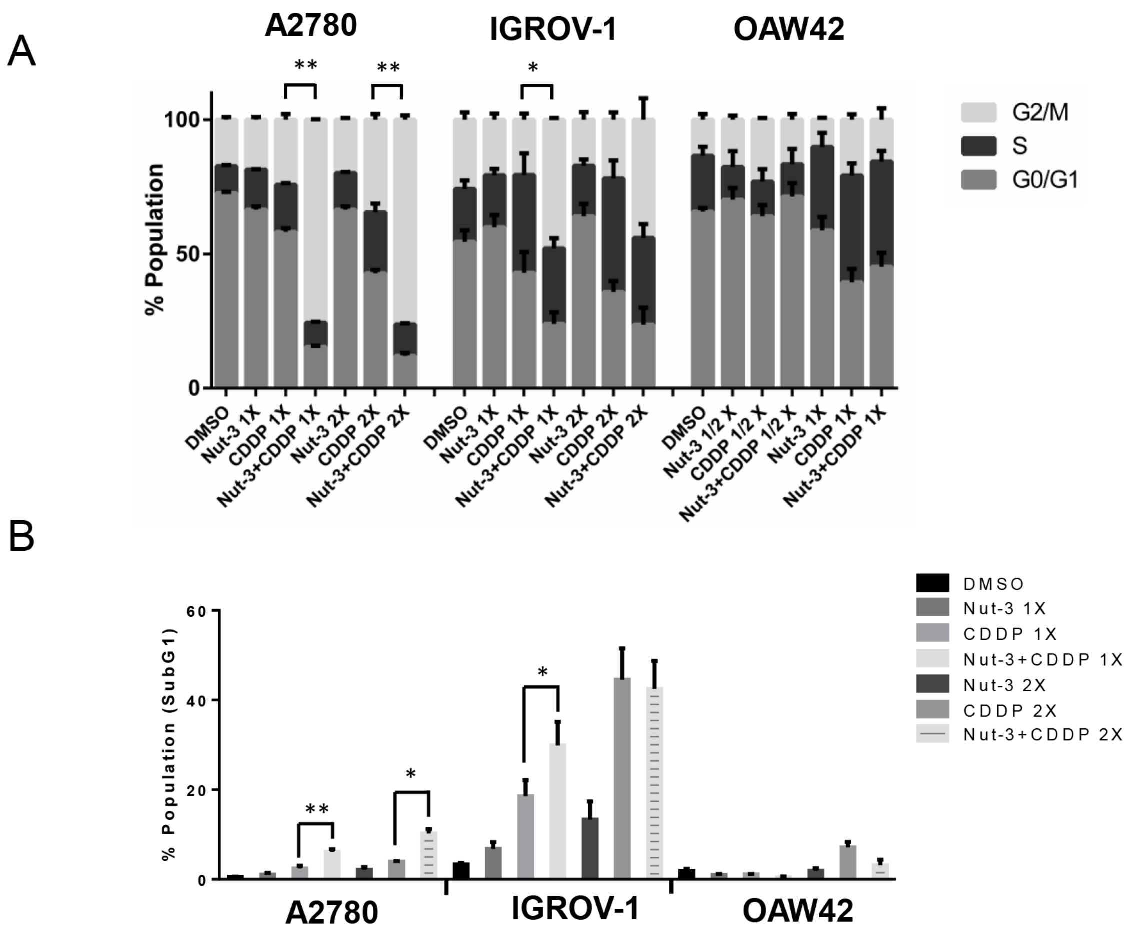

Supplementary Figure S2: Combination of Nutlin-3 with cisplatin affects the cell cycle distribution and apoptotic endpoints. Wild-type *TP53* ovarian cancer cells were treated for 72 hours with Nutlin-3 or cisplatin alone and at constant 1:1 combination ratios of 1X and 2X (1/2 X & 1X for OAW42) their respective GI_{50} concentrations. **A.** Combination of Nutlin-3 with cisplatin led to an increased proportion of cells in G2/M phase compared to either agent alone in A2780 and IGROV-1 cell lines and **B.** FACS analysis for Sub-G1 events. Nut-3, Nutlin-3; CDDP, cisplatin; *, $p < 0.05$; **, $P < 0.01$. Data are shown as the average of at least 3 independent experiments and error bars represent SEM. Statistically significant results were only shown in comparison with cisplatin on its own.

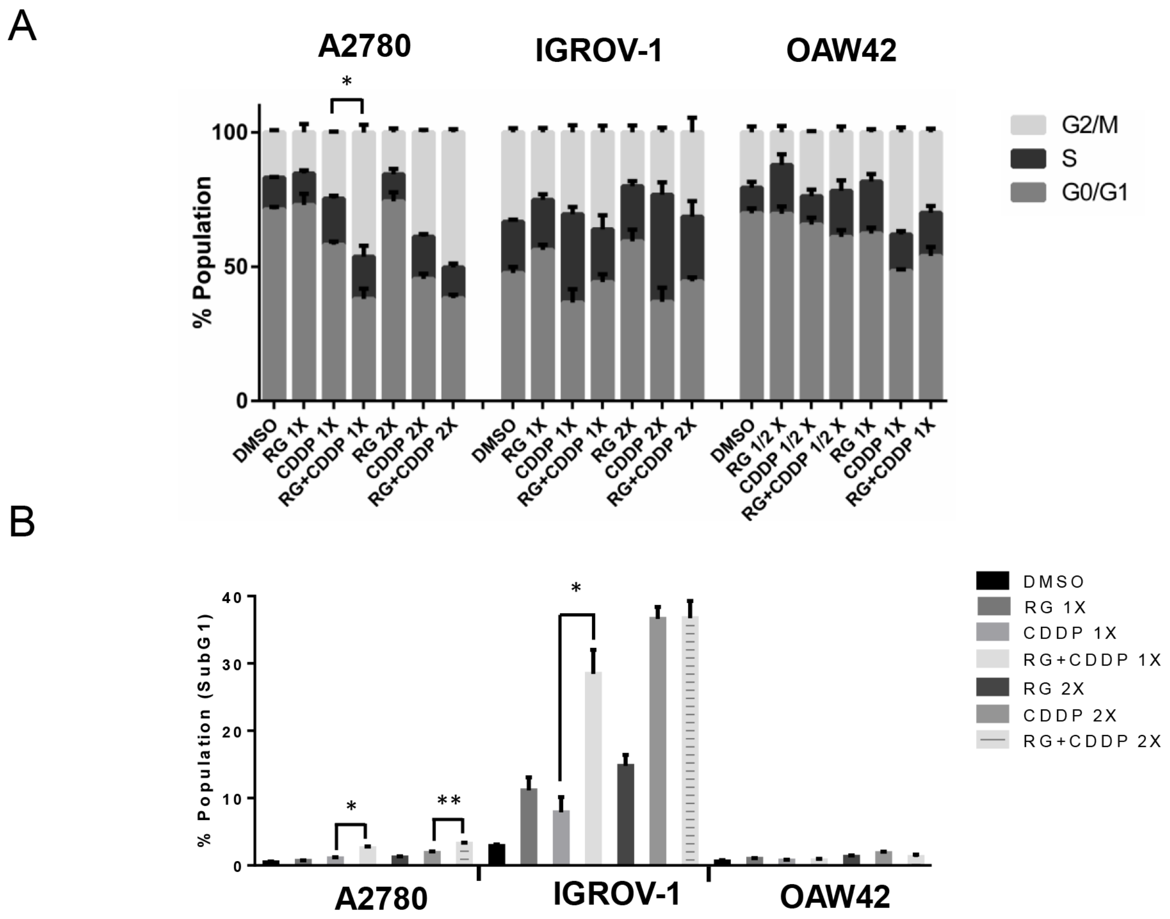

Supplementary Figure S3: Combination of RG7388 with cisplatin affects the cell cycle distribution and apoptotic endpoints. Wild-type *TP53* ovarian cancer cells were treated for 48 hours with RG7388 or cisplatin alone and at constant 1:1 combination ratios of 1X and 2X (1/2 X & 1X for OAW42) their respective GI_{50} concentrations. **A.** Combination of RG7388 with cisplatin led to increased G2/M cell cycle arrest in A2780 and IGROV-1 cell lines and **B.** FACS analysis for Sub-G1 events. RG, RG7388; CDDP, cisplatin; *, p < 0.05; **, P < 0.01. Data are shown as the average of at least 3 independent experiments and error bars represent SEM. Statistically significant results were only shown in comparison with cisplatin on its own.

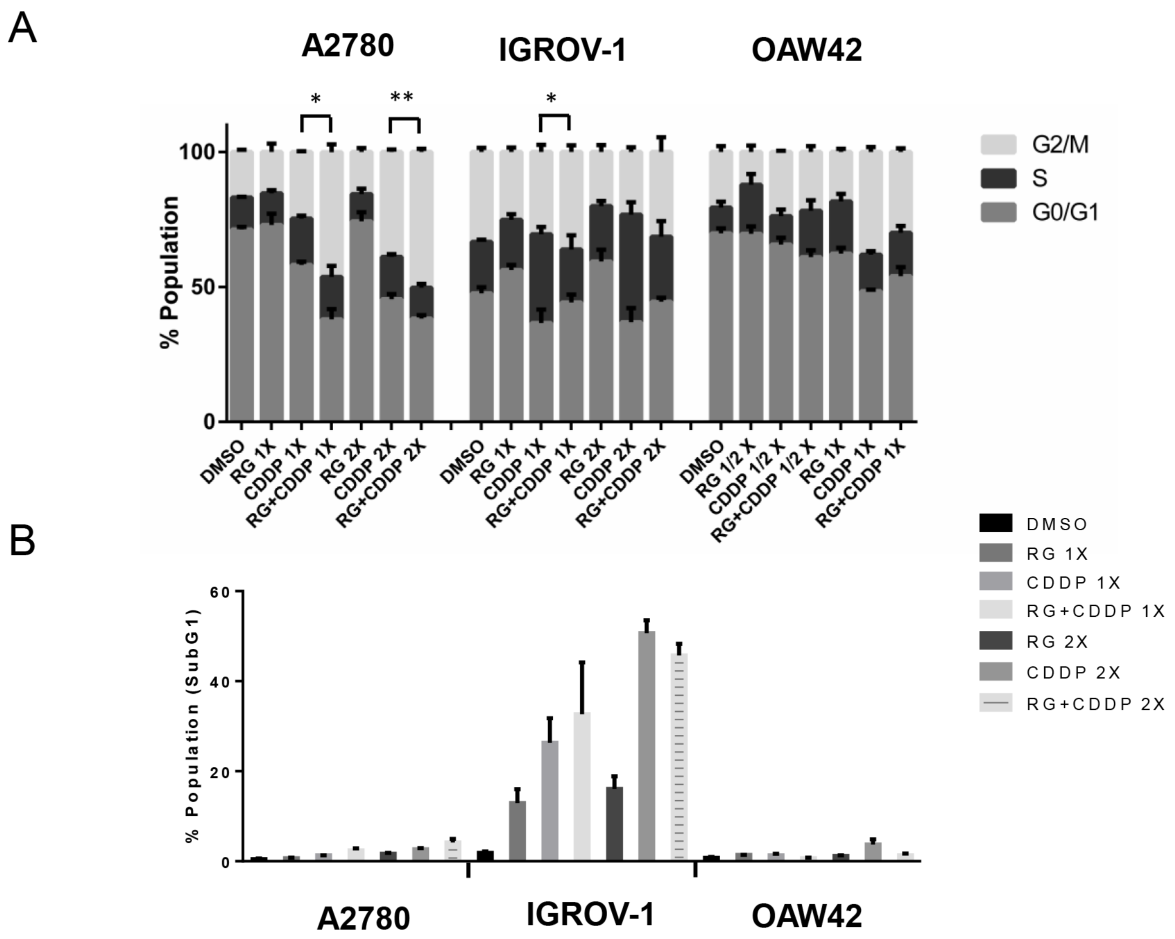

Supplementary Figure S4: Combination of RG7388 with cisplatin affects the cell cycle distribution and apoptotic endpoints. Wild-type *TP53* ovarian cancer cells were treated for 72 hours with RG7388 or cisplatin alone and at constant 1:1 combination ratios of 1X and 2X (1/2 X & 1X for OAW42) their respective GI_{50} concentrations. **A.** Combination of RG7388 with cisplatin led to increased G2/M cell cycle arrest in A2780 and IGROV-1 cell lines and **B.** FACS analysis for Sub-G1 events. RG, RG7388; CDDP, cisplatin; *, $p < 0.05$; **, $P < 0.01$. Data are shown as the average of at least 3 independent experiments and error bars represent SEM. Statistically significant results were only shown in comparison with cisplatin on its own.

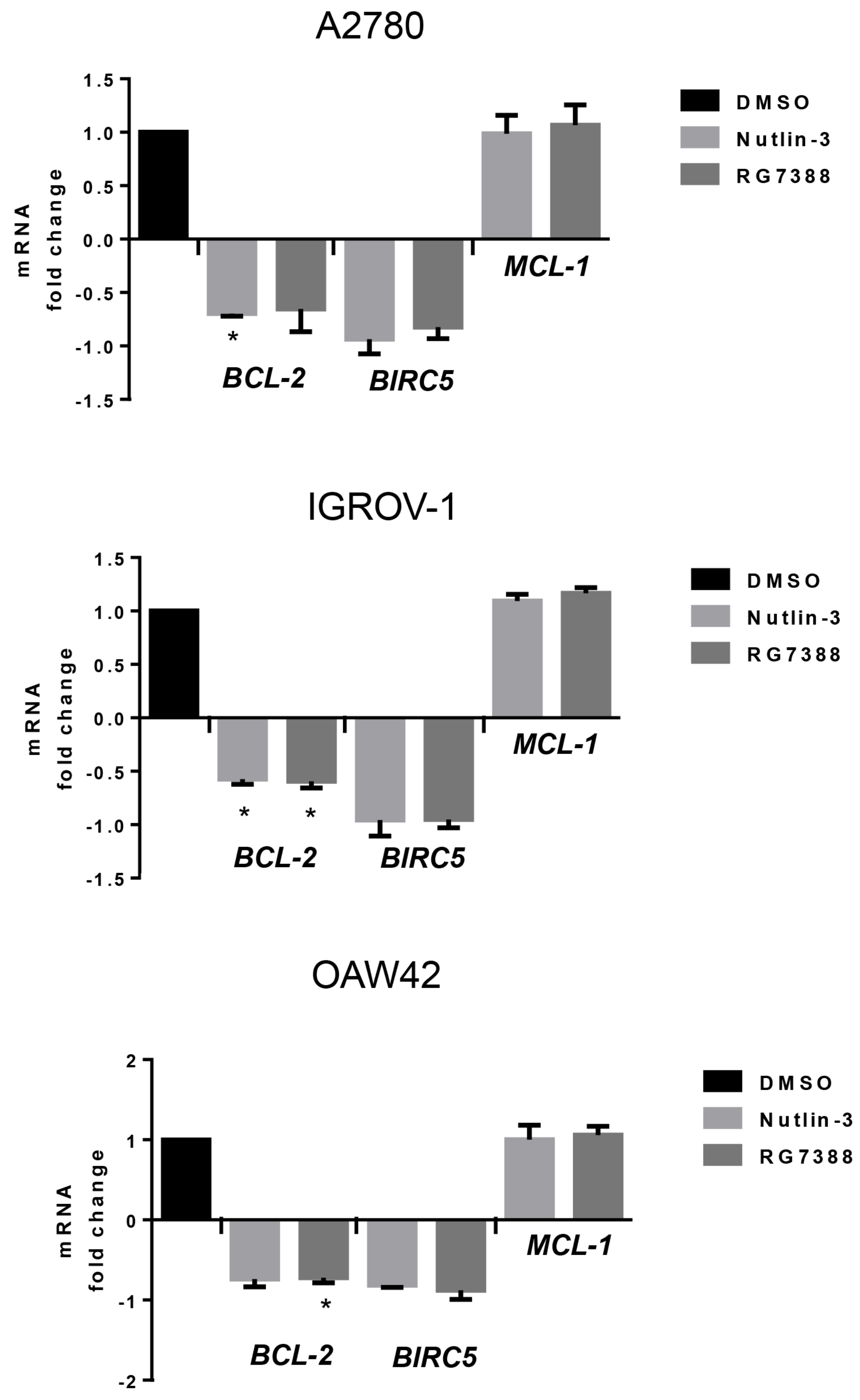

Supplementary Figure S5: mRNA expression of anti-apoptotic genes in response to 5 µM Nutlin-3 or 0.5 µM RG7388 for 6 hours relative to DMSO solvent control. *, $p < 0.05$; **, $p < 0.01$. Data are presented as mean ± standard error of mean (SEM) of three independent repeats.

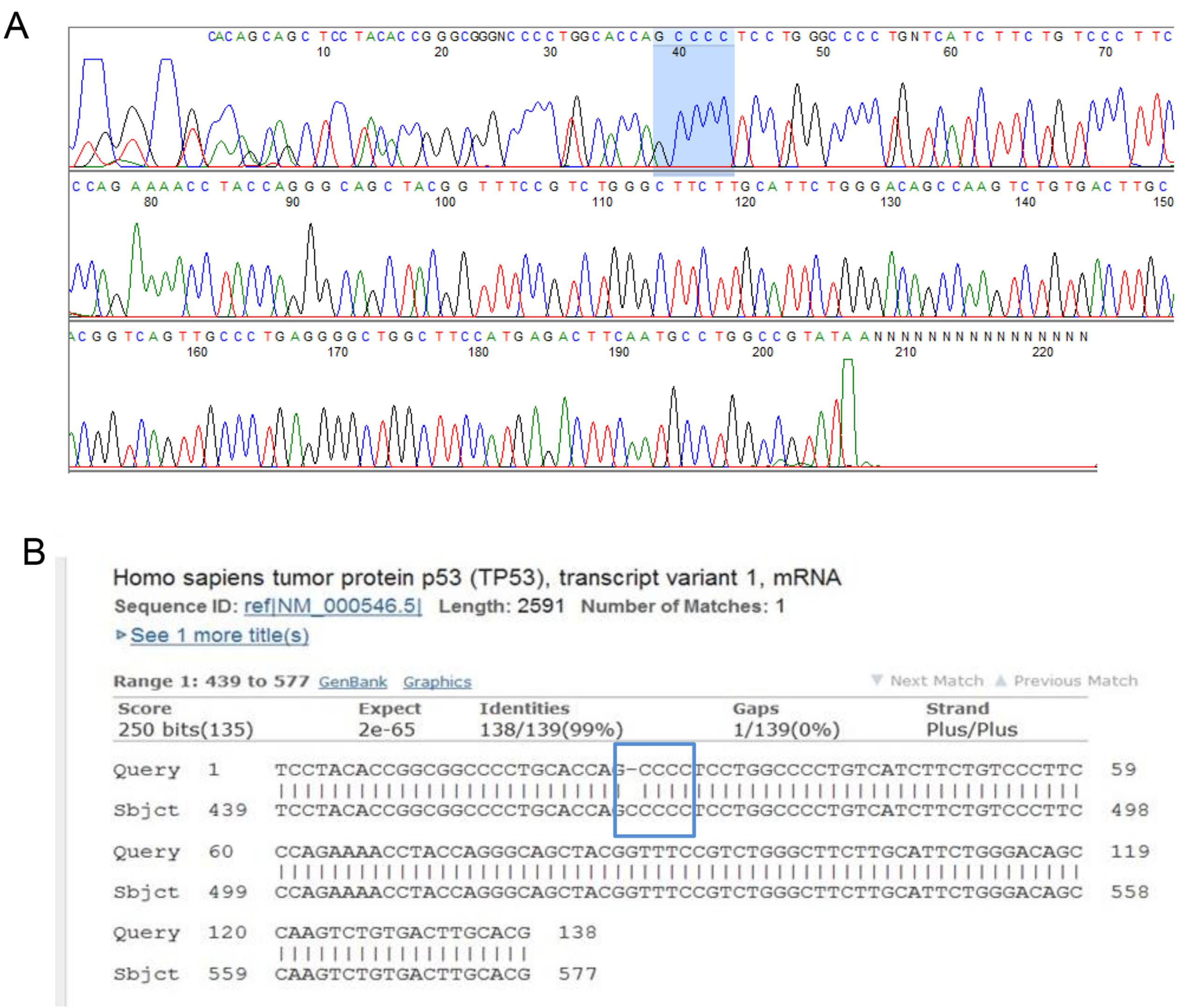

Supplementary Figure S6: Exon4 DNA sequencing of the SKOV-3 cell line. A. Codon 89 of Exon 4 Cytosine deletion, frame shift (c.265delC, P.pro89fsX33). **B.** The results of NCBI blast sequence alignment highlighting the deletion.

Supplementary Table S1: The primers and their sequences used for qRT PCR experiments for the pro-apoptotic, anti-apoptotic and cell cycle arrest genes

Gene Symbol	Target Gene Product	Primer sequence 5'-3'
AEN	Apoptosis enhancing nuclease	F-CTTCCAGGCGCTCAAGTATGT<br>R-GGGCCAGGTCCTTTAGAGAGA
BAX	BCL-2 associated X protein	F-CCCGAGAGGTCTTTTTCCGAG<br>R-CCAGCCCATGATGGTTCTGAT
BBC3 (PUMA)	BCL2 binding component 3	F-GCCAGATTTGTGAGACAAGAGG<br>R-CAGGCACCTAATTGGGCTC
TNFRSF10B	Tumor necrosis factor receptor superfamily, member 10b	F-ATGGAACAACGGGGACAGAAC<br>R-CTGCTGGGGAGCTAGGTCT
TP53INP1	Tumor protein p53 inducible nuclear protein 1	F-TCTTGAGTGCTTGGCTGATACA<br>R-GGTGGGGTGATAAACCAGCTC
MDM2	Mouse double minute 2 homolog	F-AGTAGCAGTGAATCTACAGGGA<br>R-CTGATCCAACCAATCACCTGAAT
BCL-2	B-Cell CLL/Lymphoma 2	F-GGTGGGGTCATGTGTGTGG<br>R-CGGTTCAGGTACTCAGTCATCC
BIRC5	Baculoviral IAP Repeat-Containing 5	F-AGGACCACCGCATCTCTACAT<br>R-AAGTCTGGCTCGTTCTCAGTG
MCL-1	Myeloid Cell Leukemia 1	F-GTGCCTTTGTGGCTAAACACT<br>R-AGTCCCGTTTTGTCCTTACGA
CDKN1A	Cyclin-dependent kinase inhibitor 1A (p21, Cip1)	F-TGTCCGTCAGAACCCATGC<br>R-AAAGTCGAAGTTCCATCGCTC
SESN1	Sestrin 1	F-TGCTTTGGGCCGTTTGGATAA<br>R-TGTAGTGACGATAATGTAGGGGT
GADD45A	Growth Arrest And DNA-Damage-Inducible	F-GAGAGCAGAAGACCGAAAGGA<br>R-CAGTGATCGTGCGCTGACT

Supplementary Table S2: The primers and their sequences used for qRT PCR experiments for DNA repair genes implicated in the repair of cisplatin-induced DNA damage

Gene Symbol	Target Gene Product	Primer sequence 5'-3'
DDB2	Damage-specific DNA binding protein2	F-ACCTCCGAGATTGTATTACGCC<br>R-TCACATCTTCTGGTAGGAC
ERCC1	Excision repair cross-complementing rodent repair deficiency, complementation group 1	F-CTACGCCGAATATGCCATCTC<br>R-GTACGGGATTGCCCCTCTG
MLH1	MutL homolog 1, colon cancer, nonpolyposis type 2 (E. coli)	F-GCAAACCCCTGTCCAGTCAG<br>R-CTGGGAGTTCAAGCATCTCCT
MSH2	mutS homolog 2, colon cancer, nonpolyposis type 1 (E. coli)	F-CACTGTCTGCGGTAATCAAGT<br>R-CTCTGACTGCTGCAATATCCAAT
RAD51	RAD51 homolog (S. cerevisiae)	F-CAACCCATTTCACGGTTAGAGC<br>R-TTCTTTGGCGCATAGGCAACA
RRM2B	Ribonucleotide reductase M2B (*TP53* inducible)	F-ATTGGGCCTTGCGATGGATAG<br>R-GAGTCCTGGCATAAGACCTCT
TP53BP1	Tumor protein p53 binding protein 1	F-TGAGCAGTTACCTCAGCCAAA<br>R-AAGGGAATGTGTAGTATTGCCTG
XPC	xeroderma pigmentosum, complementation group C	F-CATCGTGGGAGCCATCGTAAG<br>R-CTCACCATCGCTGCACATTTT
